# Evaluation of Inulin Replacing Chitosan in a Polyurethane/Polysaccharide Material for Pb^2+^ Removal

**DOI:** 10.3390/molecules22122093

**Published:** 2017-11-29

**Authors:** Angel Ramon Hernández-Martínez, Gustavo A. Molina, Luis Fernando Jiménez-Hernández, Adrian Hendrik Oskam, Gerardo Fonseca, Miriam Estevez

**Affiliations:** 1 Centro de Física Aplicada y Tecnología Avanzada (CFATA), Universidad Nacional Autónoma de México, Blvd. Juriquilla 3000, Querétaro C.P. 76230, Mexico; angel.ramon.hernandez@gmail.com (A.R.H.-M.); aov@fata.unam.mx (A.H.O.); gerardo@fata.unam.mx (G.F.); 2 Posgrado en Ciencia e Ingeniería de Materiales (PCeIM), Centro de Física Aplicada y Tecnología Avanzada (CFATA), Universidad Nacional Autónoma de México, Blvd. Juriquilla 3000, Querétaro C.P. 76230, Mexico; gustavomolina21@gmail.com; 3 Licenciatura en Tecnología, Centro de Física Aplicada y Tecnología Avanzada (CFATA), Universidad Nacional Autónoma de México, Blvd. Juriquilla 3000, Querétaro C.P. 76230, Mexico; luisfernandojimherz@hotmail.com

**Keywords:** heavy metals, lead pollution, wastewater treatment, composite, polysaccharide

## Abstract

Downstream waste from industry and other industrial processes could increase concentration of heavy metals in water. These pollutants are commonly removed by adsorption because it is an effective and economical method. Previously, we reported adsorption capacity of a chitosan/polyurethane/titanium dioxide (TiO_2_) composite for three ions in a dynamic wastewater system. There, increasing the chitosan concentration in composite increased the cation removal as well; however, for ratios higher than 50% of chitosan/TiO_2_, the manufacturing cost increased significantly. In this work, we address the manufacturing cost problem by proposing a new formulation of the composite. Our hypothesis is that inulin could replace chitosan in the composite formulation, either wholly or in part. In this exploratory research, three blends were prepared with a polyurethane matrix using inulin or/and chitosan. Adsorption was evaluated using a colorimetric method and the Langmuir and Freundlich models. Fourier-transform infrared spectroscopy (FTIR) spectra, scanning electron microscopy (SEM) micrographs, differential scanning calorimetry and thermogravimetric analysis curves were obtained to characterize blends. Results indicate that blends are suitable for toxic materials removal (specifically lead II, Pb^2+^). Material characterization indicates that polysaccharides were distributed in polyurethane’s external part, thus improving adsorption. Thermal degradation of materials was found above 200 °C. Comparing the blends data, inulin could replace chitosan in part and thereby improve the cost efficiency and scalability of the production process of the polyurethane based-adsorbent. Further research with different inulin/chitosan ratios in the adsorbent and experiments with a dynamic system are justified.

## 1. Introduction

Heavy metals are harmful for living beings due to their long-term environmental persistence, i.e., they cannot be decomposed by microorganisms and their toxicity persists in plants, animals and humans [1,2]. The downstream industry wastes could lead to increased concentration of heavy metals in water, air and soil. For example, mining, painting, fertilizer, textile, paper, and petroleum refining industries discharge metals into water streams [3]. The regulations adopted in several countries established the maximum levels of heavy metals discharge or emissions [4]; nevertheless, there is still a high concentration of these pollutants in the environment [3,5]. Several techniques had been proposed to remove heavy metals like chemical precipitation, membrane filtration, ion exchange, coagulation-flocculation, adsorption and electrochemical methods and additionally, modelling techniques have recently been developed which, together with the aforementioned techniques, can help improve the results obtained up to today [6,7,8,9,10,11].

In wastewater treatment, the methods most frequently used are ion-exchange, adsorption and membrane filtration [12]. The development and cost of these methods are important variables if they are used at industry scale [13,14,15]. Adsorption is an effective, economic and widely used method; it has great perspectives to pilot plant scale because its process is flexible in design and operation; additionally, the adsorbent could be regenerated [12]. Among adsorbents proposed, chitosan is a common choice; this compound is a linear polysaccharide composed of β-(1,4)-linked d-glucosamine (deacetylated unit) and *N*-acetyl-d-glucosamine (acetylated unit). The chitosan is obtained from chitin, a structural element in the exoskeleton of crustaceans, and its adsorption capacity is affected by the pH of the medium; in consequence, chitosan chemical modifications had been made [14,16,17]. Composites containing chitosan also had been widely studied, and they had proved to be efficient in heavy metals removal [18,19,20,21,22].

In a previous study, we reported the adsorption capacity of a novel composite of chitosan/polyurethane/TiO_2_ for removing Pb (II), Cd (II) and Al (III) ions in a dynamic wastewater system [18]. In that report, it was shown that a higher concentration of chitosan provides higher percentages of cation removal, but ratios higher than 50% of chitosan in relation to TiO_2_ could increase composite manufacturing costs. In this article, we address manufacturing cost problem by proposing a new formulation of the composite. Our hypothesis is that a polysaccharide of lower commercial cost like inulin food grade, could replace the chitosan in the composite formulation, either wholly or in part. Currently, inulin and its derivatives had been used for effluent treatment as flocculant, but not as part of a composite or blend for wastewater treatment [23,24].

Inulin is a fructose polymer and could be produced by a host of microorganisms. It is a broadly found in nature as a storage carbohydrate and is a Generally Recognized as Safe (GRAS) substance. Commercially, inulin is produced from chicory and where is it moderately dissolved in water, which enables its addition in aqueous medium without any precipitation [25,26,27]. Therefore, we prepared three blends: polyurethane/inulin (PI), polyurethane/chitosan (PC), and polyurethane/chitosan/inulin (PCI) and we evaluated adsorption.

For eliminating competition and selectivity between cations, we decided to use lead (II) because in Argüello’s report [12] it had the highest removal percentage in most of the analyses and it is considered one of the most toxic heavy metals because it could cause damage to the central nervous system and repercussions on liver, kidney and reproductive system [12,28,29]. In other words, our aim in this article is to provide an alternative preparation of blends with the same lead removal rates than our first effort but maintaining a relatively low production cost [18,29].

## 2. Results and Discussion

### 2.1. Blend Characterization

**Figure 1:**
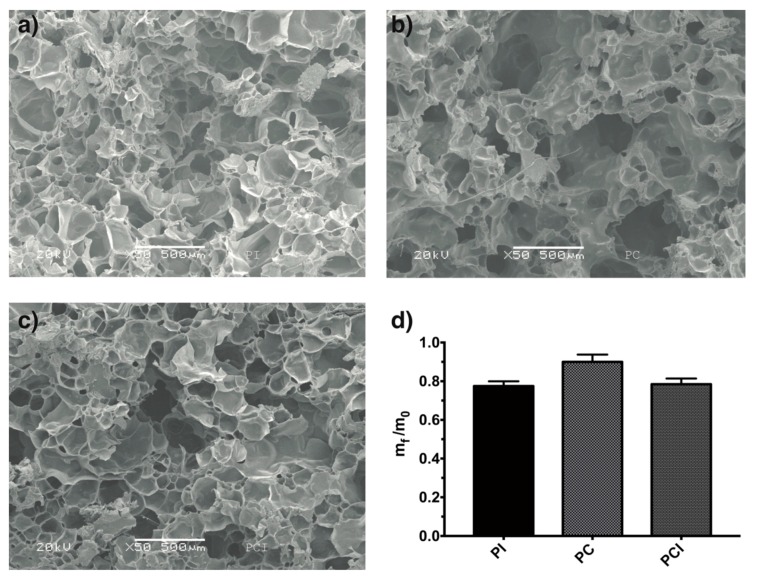
Scanning Electron Microscopy SEM images at 50× for blends materials; (**a**) blend with inulin; (**b**) blend with chitosan and (**c**) blend inulin/chitosan, all samples were synthesized at 353.15 K; and (**d**) weight loss on mechanical stirring testaccording ASTM D-2042 norm.

The morphology of prepared materials is shown in the SEM micrographs presented in Figure 1; for PI material (matrix/inulin), an average pore size of 85.70 ± 30.57 µm is seen with well-defined pores interconnected—allowing metal solution to flow throughout the pores—with an average pore size of 127.92 ± 65 µm for PC material (matrix/chitosan). In this case pores are bigger than in the PI material and also are interconnected but with a different pore structure. PCI (matrix/inulin-chitosan) was observed to have an average pore size of 95.36 ± 35.91 µm and its structure is similar to PI because the presence of inulin generates more and smaller pores. This could be explained by the interaction between the OH groups (from polysaccharides) and carbon dioxide (CO_2_) forming the polyurethane reticulation, which is generated faster in the presence of inulin (because of the hydroxyl groups in the molecule) than with chitosan. Then, more CO_2_ is present in a shorter time forming bubbles and growth sites, resulting in more and smaller pores.

**Scheme 1:**
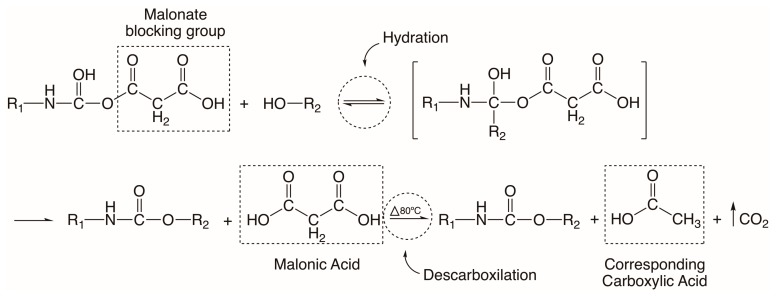
Reaction scheme for the polyurethane reaction. This schematic is based on reaction mechanisms descriptions for blocked mono-component polyurethane reported by Radice, et al. [30].

As mentioned earlier, during the mash-forming process there was a reticulation process of polyurethane due to the presence of OH– groups in the environment; the process starts a chemical reaction with the malonate-blocked polyisocyanate producing CO_2_ as a byproduct. This last compound provides volume for bubble expansion forming rigid open or closed cells (as seen Scheme 1). 

Mechanical properties are important to indicate if the material can withstand against filtration stresses; therefore, a test under a modification of ASTM D-2042 norm was conducted. Results are shown in Figure 1d, where the minimum weight loss was 10.04% for PC material; but PI and PCI materials had similar weight losses of 21.56% and 20.59% respectively. The weight loss is higher in composites with inulin than in composites with chitosan. This is explained by the electrostatic interaction between the functional groups of adsorbent components and matrix, if the electrostatic interaction is higher, the weight loss decreases. The amine group (from chitosan) has a higher electrostatic interaction than the hydroxyl group (from inulin) with the carbonyl group (polyurethane). The weight loss recorded in each sample was solid material (fine powder), which precipitated after 52 h and was recovered by decantation. The test was performed repeated twice and samples weight remained constant, none material detachment was detected. Weight loss in the first experiment was not due to washing process, because was more than 10%, but, blends are suitable to use in wastewater treatment because their weight loss was less than 60% under agitation processes, which is very similar to normal adsorption conditions.

**Figure 2:**
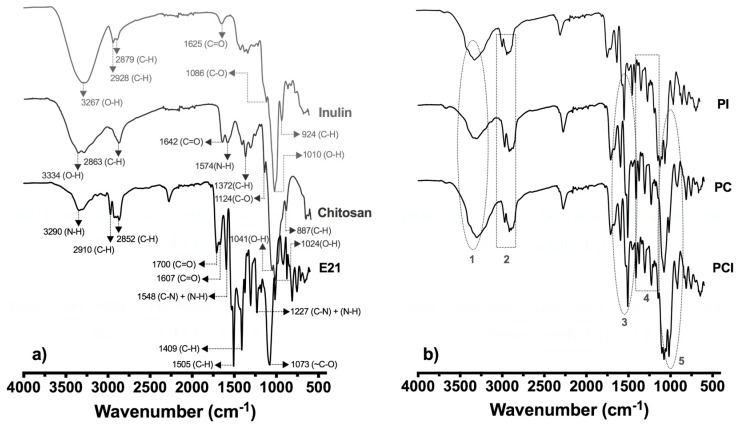
(**a**) Fourier Transform Infrared Spectroscopy (FTIR) spectra of the pure components used for manufacturing the materials; (**b**) FTIR spectra of blends components. PI = polyurethane/inulin, PC = polyurethane/chitosan, and PCI = polyurethane/chitosan/inulin.

Figure 2a shows the FTIR spectra of pure components used for manufacturing the materials. The characteristic absorption bands for polyurethane were located at 3290 cm^−1^ (N–H symmetric stretching), 2910 and 2852 cm^−1^ (CH_2_ symmetric stretching), 1700 and 1607 cm^−1^ (C=O polyurethane group), 1548 and 1227 cm^−1^ (C–N symmetric stretch & N–H deformation vibration of C–N–H), 1505 and 1409 cm^−1^ (C–H bending vibration of CH_2_) and 1073 cm^−1^ (C–O). Additionally, characteristic absorption bands of inulin were found at 3267 cm^−1^ (O–H polymeric stretch), 2928 and 2879 cm^−1^ (CH_2_ symmetric vibration), 1625 cm^−1^ (C=O stretching from carbonyl band indicating possible acetylation), 1086 cm^−1^ (C–O stretching of ring ether), 1010 cm^−1^ (OH bending vibration), 924 cm^−1^ (CH_2_ twisting vibration). Finally, absorption bands of chitosan were shown at 3384 cm^−1^ (O–H stretching vibration), 2863 cm^−1^ (CH_2_ symmetric vibration), 1642 cm^−1^ (C=O stretching in secondary amide), 1574 cm^−1^ (N–H bending vibration of NH_2_ groups), 1372 cm^−1^ (C–H bending vibration of CH_2_), 1124 cm^−1^ (C–O stretching of ring ether), 1041 and 1024 cm^−1^ (O–H bending vibration), 887 cm^−1^ (CH_2_ twisting vibration).

In Figure 2b, five different zones were identified; it could be seen that from zone 2 to 4, the characteristic absorption peaks of polyurethane could be clearly identified—this is because the synthesis method is only based on electrostatic interaction between the polysaccharide and the polyurethane. In region 1, it was identified a change in intensity of absorption peak corresponding to O–H. The highest peaks are shown in samples containing inulin while the ones with chitosan were reduced, but in region 5 the O–H peaks overlap with the C–O–C peak indicating, in all samples, that the polysaccharide is distributed in the most external part of the polyurethane resin which is extremely favorable for adsorption (suggested because through ATR-FTIR we obtained blends surface data), also these peaks are similar to the corresponding polysaccharide used. In the case of the PCI material, inulin is more exposed to the surface than chitosan.

**Figure 3:**
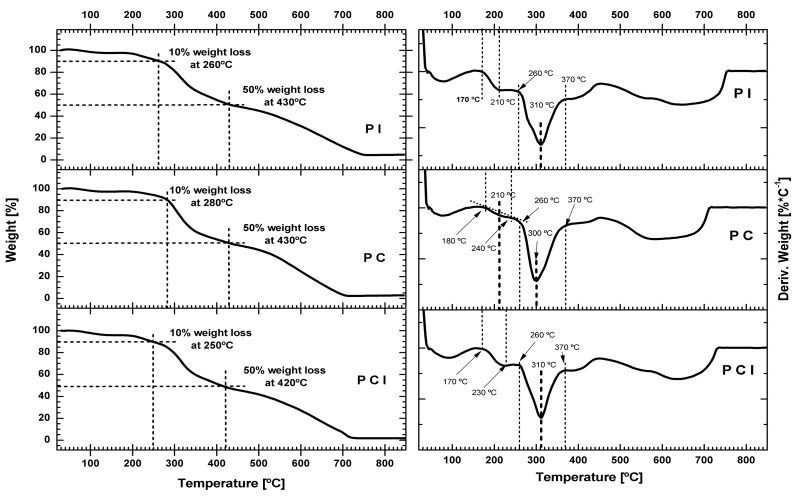
On the right; TG curves of the thermal degradation and on the left; DTG curves of the thermal degradation of blends.

Figure 3 exhibits the curves obtained by the thermogravimetric analyzer; on the right, the thermal degradation values (TG) are plotted and on the left the derivated data (DTG) are plotted. These data corresponds to thermal degradation of blends in air at 10 °C heating rates. It can be seen that the TG curve has three weight-loss steps and the DTG curve (Figure 3; on the left) also has three inflections. It is observed that no significant degradation occurred before 200 °C for all blends; between 80 and 150 °C the weight loss was 5%, which could be attributed to moisture within the material. At temperatures above 200 °C, it was observed the beginning of oxidative degradation of the materials; for example the blend with inulin had a weight loss of 10% around 260 °C; and the same weight loss was around 280 °C and 260 °C for blends with chitosan and chitosan/inulin respectively (see Tg curves, Figure 3—right side).Hence, this behavior on the thermal degradation of the blends is due to the incorporation of inulin in the blends in this way, for the material without inulin its thermal degradation behavior (weight loss around 280 °C) is totally attributable to the chitosan, and the thermal degradation behavior (weight loss around 260 °C) of the two other materials is explained by the thermal decomposition of the inulin incorporated in each one. 

The polysaccharide decomposition in the blend could cause the weight loss in the first stage. From the DTG curve, the inulin degradation was completed in a single stage within temperature range between 180 °C and 240 °C; similarly, chitosan and chitosan/inulin degradations were completed between 170 °C and 230 °C, these results are consistent with data reported previously [31,32,33]. Between 420 and 430 °C, the materials lose 50% of their initial weight and in the DTG curve maximum degradation was found at around 310 °C.

Typical thermal behavior for rigid polyurethanes reported by other authors [34,35,36,37] was observed around this temperature and up to the last degradation temperature of the blends. As mentioned in the methods section, the TG analysis was simultaneously obtained with differential scanning calorimetry; in this way it was possible to corroborate that the stages of weight loss corresponded to an oxidative degradation because exothermic processes were observed.

### 2.2. Removal Experiments

**Figure 4:**
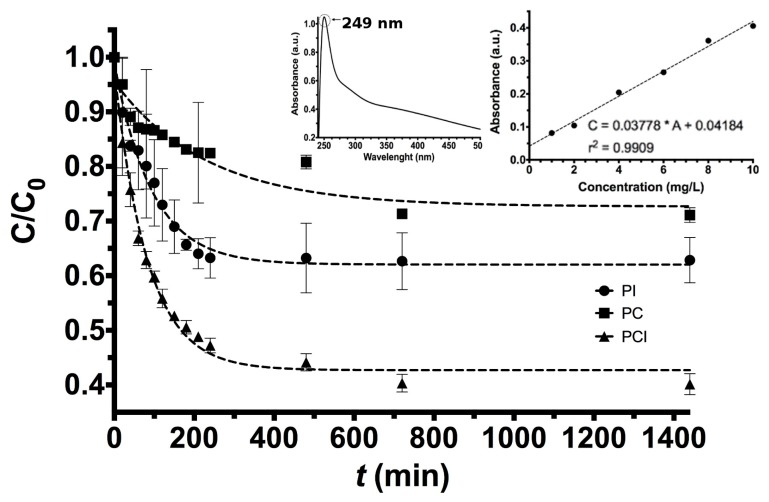
Removal conditions of Pb(II) ions by 192 mg of PC, PI or PCI material using λ = 249 nm as maximum absorbance at pH = 7.8 and 25 °C. C = ion Lead concentration; C_0_ = Pb^2+^ initial concentration, 10 mg/L; C/C_0_ = Pb^2+^ fractional removal; t = contact time between material and lead solution.

First, it was necessary to determine the characteristic maximum absorption, calibration curve and equilibrium time, which were determined as shown in Figure 4. Here it could be seen that results followed the Beer–Lambert law and were reliable (*r*^2^ = 0.9909) at λ = 249 nm, also were within Pb^2+^ concentrations between 1 and 10 mg L^−1^. Additionally, Figure 4 clearly shows that for 720 min (12 h), the amount of Pb^2+^ cations removed by 192 mg of dried materials into 10 mL of the Pb^2+^ solution remains constant. 

Therefore, all further removal experiments were conducted for 12 h as absorption time, with 192 mg of dried blends into 10 mL of the Pb^2+^ solution at initial concentration of 10 mg/L; with approximately initial pH = 8.0. 

**Figure 5:**
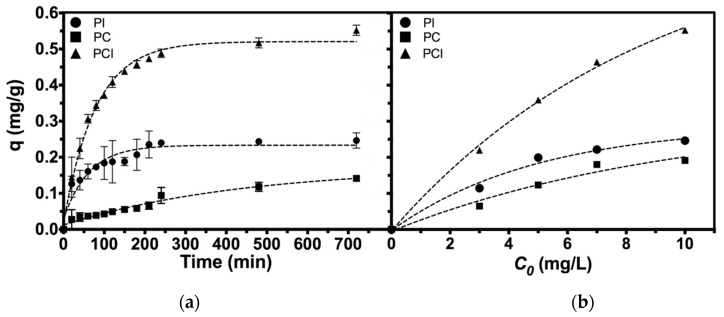
(**a**) Time-dependent adsorption capacity at 25 °C; (**b**) Adsorption capacity, varying initial concentration of lead II(Pb^2+^).

Figure 5 shows the adsorption capacity (q) versus time and variable initial concentration of Pb^2+^. In all cases the conditions are those described in the previous section, unless otherwise indicated. 

**Scheme 2:**
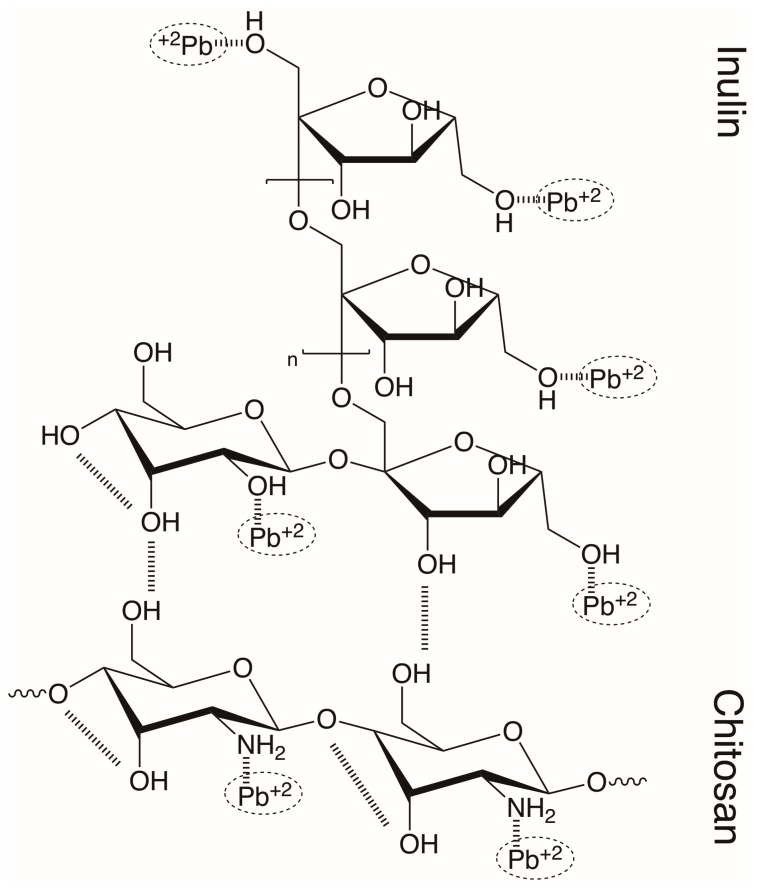
Possible interaction between inulin-chitosan and Pb(II).

The capacity of Pb^2+^ uptake by all materials at different contact time is shown on Figure 5a. For all samples, lead is completely adsorbed at 12 h, but lead uptake increased rapidly in the first 3 h with inulin adsorbents due to a larger amount of the OH group present in it. This is an effective factor in a batch process. Lead has high tendency to form oxygen and nitrogen groups according to the hard and soft acids and bases theory, in that sense it is possible to explain the lead adsorption by complexation between oxygen atoms (vacant active) from matrix/inulin with lead ions) [37,38,39]. The vacant active sites at the beginning of the adsorption process increase lead uptake, then with more contact time, the rate of lead uptake decreases because of active sites occupation. As a consequence, the active sites of inulin were occupied in less time than the active sites of chitosan (as seen Scheme 2). 

On Figure 5b, results varying adsorbate dosage (lead cations) are shown; here, the amount of Pb^2+^ adsorption increased with an increase in the adsorbate dosage from 1 to 10 mg/L for all adsorbents. This is because with high adsorbate concentrations, the number of cations increases in the system; hence, more metal ions are available for reacting with active sites, increasing adsorption. It was expected a slower increment at the end of experiments because blends pores were close to saturation values, making the ions transportation more difficult.

### 2.3. Kinetic Models

The constant rate parameters (*k*_1_ and *k*_2_) could be calculated from the linear plots of log*(q_e_ − q_t_)* against *t* and *t*/*q_t_* against *t*; and their values are shown in Table 1. Also, the initial adsorption rate constant *k*_2*i*_ (mg·g^−1^ min^−1^) could be determined using the relationship *k*_2*i*_ = *k*_2_*q_e_*_2_ according to the pseudosecond-order kinetic adsorption [40,41,42]. 

In the pseudofirst-order kinetic model, the linear correlation coefficient (*R*^2^ = 0.9463) indicates that this model applies for PC material and *q_e_* value (*q_e,cal_*) from it, is consistent with the experimental results. On the other hand, the pseudosecond-order kinetic model has been calculated and experimental *q_e_* values for PI and PCI blends are consistent, with a correlation coefficient above 0.999. These results indicate that PC blend adsorption is controlled by mass transfer and PI and PCI processes are controlled by chemical reactions.

Additionally, the Langmuir and Freundlich parameters obtained from the plots of *C_e_*/*q_e_* against C_e_ and log (*q_e_*) against log (*C_e_*) were listed in Table 1. From here, it could be seen that the correlation coefficients (*R*^2^) of the Langmuir isotherm model for PI is really close to 1 and the maximum adsorption capacity is in accordance with the experimental value. This means that for this specific sample, the model is suitable for evaluating the adsorption isotherm. From the Freundlich isotherm model for PC the coefficient is closer to 1—a value that is suitable for this specific sample. The PCI sample is better described by Langmuir model, the value of *q_m_* is not far from the experimental value; this indicates that for this sample and PI, there is a monolayer coverage of Pb^2+^ on the surface of the blends. For the Freundlich isotherm model, it can be seen that the PC sample is not entirely heterogeneous (values of *n* are not close to 0) and the *n* value below to 1 indicates the preference for a chemical process of adsorption.

The regeneration of sorbents is one of the crucial aspects in water treatments and toxic substances removal, as it is closely related to the economy of treatment technology. 

**Figure 6:**
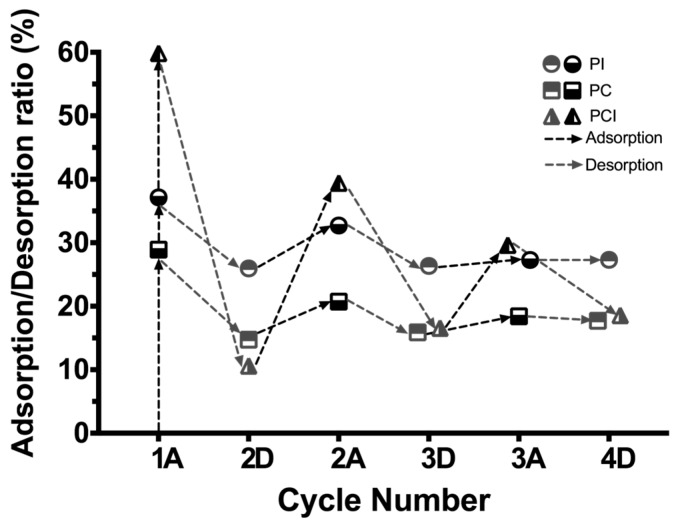
Recycling of blends; adsorption/desorption capacity for four cycles at 25 °C.

Figure 6 shows that the adsorption capacity ratio of Pb^2+^ from the initial solution (10 mg/L) decreases with increasing the adsorption–desorption cycles, whereas the values are 37.50% (PI), 28.91% (PC) and 59.86% (PCI) for the first adsorption cycle (1A number cycle) and 27.29% (PI), 18.41% (PC) and 29.64% (PCI).Also, there is a recovery ratio of Pb^2+^ from the initial solution reached 74.04% (PI), 85.28% (PC) and 89.38% (PCI) for the first desorption cycle (2D cycle number), and 72.70% (PI), 82.29% (PC) and 81.44% (PCI) for the third desorption (4D cycle number). These indicate that the blend containing chitosan has a better recovery capacity for Pb^2+^ compared to inulin, suggesting a higher preference for this specific ion to be attached to inulin. It was also noted that all the blends maintain almost the same recovery ratio with increasing cycles with a slightly loss that indicates that the metals adsorbed since the first cycle remain constant in the material and this is proved in the adsorption capacity that decreases and almost maintained a similar value resect recovery. Also, is seen that chitosan controls the recovery ratio in the blend PCI and inulin controls the adsorption ratio seeing and synergic effect of both of the polysaccharides.

## 3. Materials and Methods

### 3.1. Chemical Compounds

Chitosan (low molecular weight, 0.75–0.85 of deacetylation) (PubChem CID:21896651), lead (II) nitrate (>99%) (PubChem CID:24924), nitric acid (70%) (PubChem CID:944), citric acid (99%) (PubChem CID:311), NaOH (>99.9%) (PubChem CID:14798), and 1,2-Diaminocyclohexane-*N*,*N*,*N*,*N*-tetraacetic acid monohydrate (DACT) (PubChem CID:2723844) (for complexometry, >98%). 

All chemical compounds were provided from Sigma Aldrich Co. Mexico; the monocomponent polyurethane polymer (E21) was provided from Bayern Material Science, Germany; inulin polysaccharide (food grade) was purchased from commercial source as prebiotic from e-nature, Mexico. All experiments were carried out using deionized water (dH_2_O) and materials were used as received.

### 3.2. Blends Manufacturing Process

**Scheme 3:**
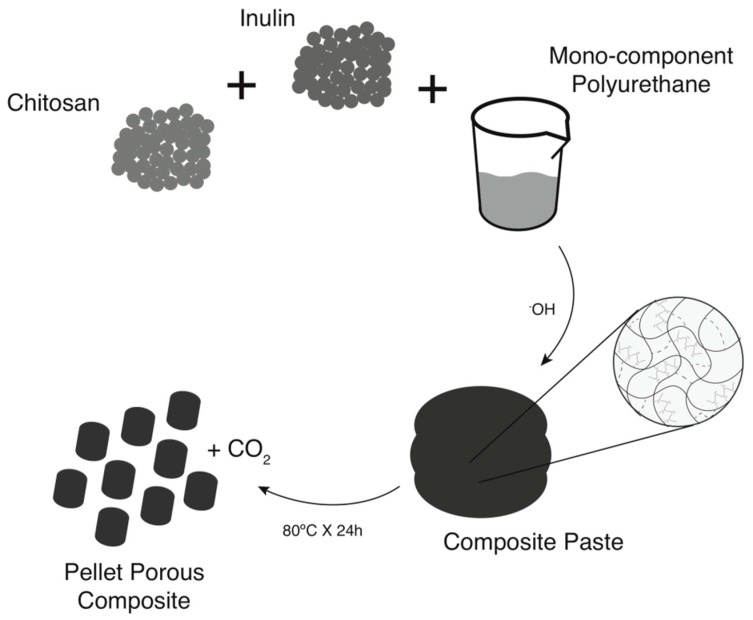
Process schematic of polyurethane-based composites manufacturing.

Three blends were prepared (as seen in Table 2) using polyurethane as the polymer matrix or polymeric structure of them; polyurethane with chitosan (PC, mass relationship 1:1); polyurethane with inulin (PI, mass relationship 1:1), and polyurethane with a chitosan-inulin blend (PCI, mass relationship 2:1:1). The chitosan, inulin and their blend were mixed and dispersed in the polyurethane until it was saturated; then, the mix was molded into rods of 4 mm diameter (as seen Scheme 3). Relative humidity was monitored using a 4040 traceable humidity monitor (Control Company) and it was maintained in 48% for all the experiments. As temperature affects the pore size composition of polyurethane [12,43], all blends were subjected to a thermal treatment at 80 °C to obtain a large number of small pores [18].

### 3.3. Characterization and Methods

The surface morphology was obtained using a scanning electron microscope (SEM) Jeol JSM-6060LV operated at 20 kV in secondary electron mode with different magnifications; samples were covered with a gold film prior to observation. The pore sizes distribution data was obtained by analysis of SEM images using ImageJ software (open source).

Mechanical properties were analyzed with mechanical stirring in sealed glasses (full with water); this was performed with a modification of ASTM D-2042 norm. Blends samples were stored in water for one day, and then they were stirred at 6000 rpm in two 15 min periods with 5 min rest in between. Afterwards, samples were removed from water, dried and weighed (to determine weight lost). 

The FTIR spectroscopy characterization of blends was obtained by a Spectrum Two FTIR Spectrophotometer (Perkin Elmer). Dried samples with minimal moisture content were measured using ATR technique and spectra were collected from a wavenumber range of 400 to 4000 cm^−1^. A Thermogravimetric Analyzer (TG) with differential scanning calorimetric (DSC) capability (TGA/DSC-1) from Mettler-Toledo Inc. were used to perform simultaneous thermal analysis while heating the samples from 20 to 850 °C at the rate of 10 °C·min^−1^ in the air.

### 3.4. Removal Test

The determination of Pb^2+^ ions removed was performed by the UV spectrophotometric method developed by [44,45,46] using an UV-Vis spectrophotometer VWR-1600PC. In the concentration range between 10 mg·L^−1^ and 1 mg·L^−1^, where calibration curves obtained showed linearity, the Beer–Lambert law was obeyed. According it; an initial Pb^2+^ solution was prepared by dissolving nitric acid and lead (II) nitrate in 500 mL of dH_2_O for a 100 mg·L^−1^ concentration; then, 10 mL of this solution were diluted up to 100 mL for an initial concentration (*C*_0_) of 10 mg·L^−1^; under this condition, the initial pH was 7.8. 

Subsequently, in fifteen different vials; 10 mL of this solution were put into with 192 mg of dried blends in each one; later at scheduled times the blends were removed, pH was adjusted to 8 by dropping a concentrated solution of NaOH to avoid Pb^2+^ ions precipitation and an equimolar amount of (DACT) was added to proceed to make the measurements at UV spectrophotometer. Under these conditions, all removal tests were first conducted at room temperature (297.15 K) and then at different temperatures from 293.15 to 353.15 K in 20 degree increments. The fractional removal and adsorption capacity of the blend samples for the lead ion at given time t or equilibrium were calculated by the following equations respectively:

(1) $$r=\frac{C}{C_{0}}$$

(2) $$q=\frac{V*\left(C_{0}-C\right)}{m}$$

where *r* is the retention rate of Pb^2+^ considering total concentration as 1, *q* is the adsorption of Pb^2+^ (mg/g) at a given time or equilibrium (*q_t_* or *q_e_*), V is the volume of Pb^2+^ solution used (mL), *C*_0_ and *C* are the initial and final concentrations (mg·L^−1^) and m is the mass of the blend used (mg). In all cases, three parallel measurements were performed to obtain a maximum standard deviation of 10%.

Finally for the regeneration study; Pb(II)-adsorbed on the blends was eluted with 0.1 M HCl solution, under magnetic stirring for 24 h, then the blends were regenerated using an 0.1 M NaOH solution and washed with dH_2_O to remove adsorbed alkali and finally dried at 70 °C in order to be reused. The concentration of Pb(II) desorbed into the solution was analyzed using the same UV spectrophotometric method described before using the complex DACT-Pb^2+^. 

### 3.5. Theoretical Considerations

A pseudo first- and second-order kinetic model equations are used to evaluate experimental data from batch Pb^2+^ removal [47]. The pseudo first-order kinetic model was suggested by Lagergren for adsorption of solid/liquid systems and it has been widely used to describe metal adsorption kinetics and could be expressed by the following linear expression spectrophotometric method developed by [48]:

(3) $$\log\left(q_{e}-q_{t}\right)=\log\left(q_{e}\right)-\frac{k_{1}t}{2.303}$$

The pseudo second-order model was suggested by Ho and McKay [48,49,50]:

(4) $$\frac{t}{q_{t}}=\frac{1}{k_{2}q_{e}^{2}}+\frac{t}{q_{e}}$$

where *q_e_* and *q_t_* (mg·g^−1^) are the adsorption capacities of the blends for Pb^2+^ removal at equilibrium and time *t*, respectively. *k*_1_ (min^−1^) is the constant rate of the pseudo first-order adsorption and *k*_2_ (g·mg^−1^·min^−1^) is the constant rate of the pseudo second-order adsorption kinetic [47,48,49,50,51].

The isotherm data is used to describe the interaction of adsorbent molecules with surface of blend, the correlation of equilibrium and accurately representation of results using either a theoretical or empirical equation is essential in evaluating adsorption by interpretation, prediction and mechanism for the specific application design. In order to do this, the Langmuir and Freundlich isotherm models had been used.

Langmuir Model: A straightforward non-linear isotherm model, it is based on the assumption of a structurally homogeneous adsorbent, where adsorption sites are identically and energetically equivalents, with no interactions between the adsorbed molecules. At low surface coverage, the isotherm model reduces to a linear relationship which can be expressed in its linear form as:

(5) $$\frac{C_{e}}{q_{e}}=\frac{1}{q_{max}b}+\frac{C_{e}}{q_{max}}$$

where *q_e_* is the amount of Pb^2+^ adsorbed at equilibrium (mg·g^−1^), *C_e_* is the liquid-phase Pb^2+^ concentration at equilibrium (mg·L^−1^), *q_max_* is the maximum adsorption capacity or saturation capacity of the adsorbent (mg·g^−1^), and *b* is the Langmuir adsorption constant (L·mg^−1^) [47,48,49,50].

Freundlich Model: An empirical equation and a widely used nonlinear adsorption equilibrium model, this model is used for adsorption on heterogeneous surfaces with a uniform energy distribution, and reversible adsorption and is not restricted to the formation of the monolayer. In other words, the adsorbent capacity could be improved increasing the concentration of the adsorbate in the medium. The equation is usually presented in the following logarithmic form:

(6) $$\log\left(q_{e}\right)=n\log\left(C_{e}\right)+\log K$$

where *K* (mg·g^−1^) is the Freundlich constant related to adsorption capacity and *n* is the heterogeneity factor related to adsorption intensity; both empirical constants depend on several environmental factors. The value of *n* is from 0 to 1 and indicates the non-linearity degree within solution concentration and the adsorption, if the n value is below 1, the adsorption is a chemical process and if it is above 1 adsorption is a physical process. The more heterogeneous the surface, the closer to 0 is the value of *n* [48,49,50,51,52].

## 4. Conclusions

Three blends were prepared for the lead (II) adsorption—PI, PC and PCI—in order to investigate the possibility to replace chitosan for inulin in a composite formulation for wastewater treatment. Their weight losses indicate that all blends prepared could be used for that treatment. Results indicate that the initial concentration of lead (II) cation influences directly the adsorption capacity. The kinetic experimental data were fitted by pseudo first- and second-order models. These results indicate that inulin improved chitosan adsorption capacity, but it did not show good results alone with the polymeric matrix. In conclusion, inulin could replace chitosan in part in the composite formulation to reduce manufacturing costs; also, the PCI (polyurethane/chitosan/inulin) blend is promising for testing other toxic elements removal. Further research must study the adsorption performance of this composite in dynamic conditions.

## Figures and Tables

**Table 1:** Kinetic calculations results for pseudo-first; second-order models; Langmuir and Freundlichmodels by materials.

Kinetic Models		Material		
		PI	PC	PCI
Pseudofirst-order model	*q_e,exp_* (mg·g^−1^)	0.2463	0.1915	0.5523
	*q_e,cal_* (mg·g^−1^)	0.1611	0.1772	0.3482
	*k*_1_ (min^−1^)	0.00957	0.00180	0.00579
	*R*^2^	0.8913	0.9463	0.8757
Pseudosecond-order model	*q_e,cal_* (mg·g^−1^)	0.2603	0.2552	0.5938
	*K*_2_ (g·mg^−1^·min^−1^)	0.10400	0.00906	0.02914
	*K*_2*i*_ (mgg^−1^·min^−1^)	0.00705	0.00059	0.01027
	*R*^2^	0.9996	0.6545	0.9992
Langmuir model	*q_m_* (mg·g^−1^)	0.2669	0..4437	0.7402
	*b* (L·mg^−1^)	1.7412	0.1393	0.1768
	*R*^2^	0.9999	0.9821	0.9665
Freundlich model	*K* (mg·g^−1^)	0.1682	0.0589	0.1505
	*n*	0.2177	0.7141	0.4882
	*R*^2^	0.9385	0.9939	0.8441

**Table 2:** Components ratios for each blend prepared.

Sample	Inulin (g)	Chitosan (g)	Polyurethane (g)
PC	0	3	3
PI	3	0	3
PCI	1.5	1.5	3
